# Caul birth: A retrospective cohort study on prevalence, predictors, and maternal and neonatal outcomes

**DOI:** 10.18332/ejm/223962

**Published:** 2026-06-30

**Authors:** Sofia Tallhage

**Affiliations:** 1 Department of Health and Caring Sciences, Linnaeus University, Kalmar, Sweden; 2 Department of Obstetrics and Gynecology, Kalmar County Hospital, Kalmar, Sweden

**Keywords:** caul birth, amniotomy, maternal outcomes, birth experience, neonatal outcomes

## Abstract

**INTRODUCTION:**

Caul birth, in which an infant is born with part of the amniotic sac covering the head or body, is a rare phenomenon with limited research on its prevalence, predictors, and outcomes in contemporary midwifery and obstetric practice. Although it has historically been attributed cultural and symbolic significance, it remains underexplored in research. The aim of this retrospective cohort study was to investigate the prevalence of caul birth, its predictors, and associated neonatal and maternal outcomes in a Swedish hospital setting.

**METHODS:**

A retrospective cohort study including all births at a Swedish hospital in 2023 (n=1382) was conducted. Data were obtained from medical records. Women with caul birth were compared with those undergoing amniotomy during labor (n=377). Multivariable logistic regression was used to identify predictors and maternal and neonatal outcomes associated with caul birth.

**RESULTS:**

Caul birth occurred in 1.5% (n=21) of births and was associated with maternal overweight according to BMI (AOR=3.42; 95% CI: 1.10–10.64), spontaneous onset of labor (AOR=18.21; 95% CI: 2.31–143.50), and multiparity (AOR=11.11; 95% CI: 1.34–92.10). Epidural analgesia (AOR=0.10; 95% CI: 0.02–0.44) and longer duration from hospital admission to birth (AOR=0.63; 95% CI: 0.48–0.84) were associated with decreased odds of caul birth. No adverse neonatal outcomes were observed.

**CONCLUSIONS:**

Caul birth was rare but associated with spontaneous, low-intervention labor and reassuring maternal and neonatal outcomes. These findings align with current recommendations against routine amniotomy in uncomplicated labor, although larger studies are needed.

## INTRODUCTION

**Figure 1:**
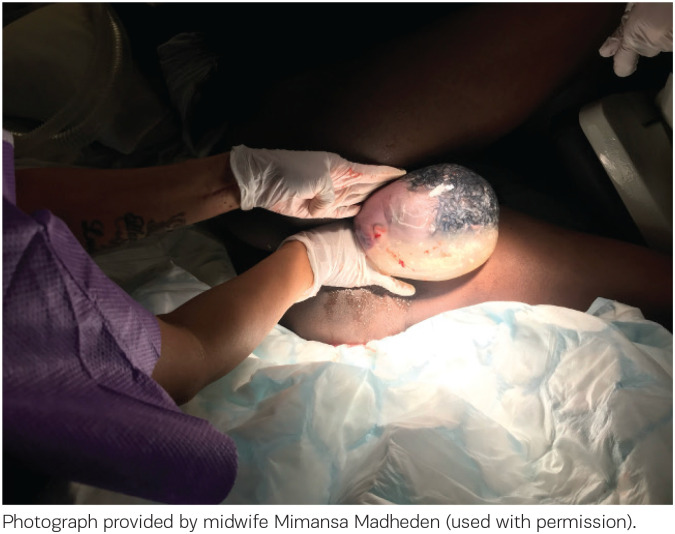
Caul birth during vaginal birth, showing a newborn with part of the amniotic membrane (caul) covering the head

**Figure 2:**
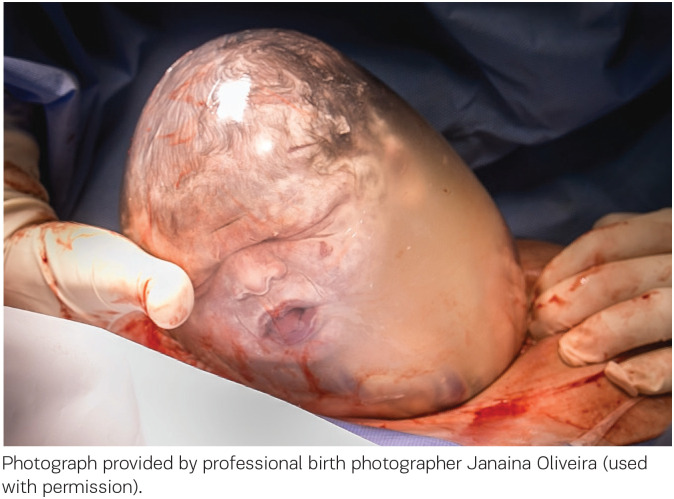
Caul birth during caesarean section, showing a newborn with intact amniotic membranes

Caul birth is a phenomenon in which remnants of the amniotic membrane remain attached to the neonate’s head or face at birth, with the membranes partially intact. In contrast, *en caul* birth occurs when the baby is delivered entirely enclosed within an intact amniotic sac. Caul birth and *en caul* birth can occur in both vaginal births (Figure 1) and cesarean sections (Figure 2)^1^. This article focuses on caul birth.

Historically, caul births have been imbued with powerful symbolic and magical significance^2,3^. In medieval times, the midwife’s role extended beyond assisting the birthing woman to interpreting particular signs and omens during labor – one of the most notable being the ‘victory cap’, known in Swedish as *segerhuva*, which reflects the enduring belief that being born in the caul signified an extraordinary future and was regarded as a sure sign of good fortune for the newborn^4^. In Swedish history, both King Karl XII and Queen Christina were reportedly born in their caul, a circumstance believed to foretell a life marked by success and destiny. Traditionally, the caul was often saved and worn as a talisman to ensure continued luck throughout life. The membranes from King Karl XII’s birth have been preserved at the Royal Armory in Stockholm’s Royal Palace. In 19th-century England and Wales, cauls were sold by midwives and were believed to protect against drowning; an 1835 London Times ad read: ‘A child’s Caul to be disposed of, a well-known preservative against drowning. Price 10 guineas’^4^.

**Figure 3:**
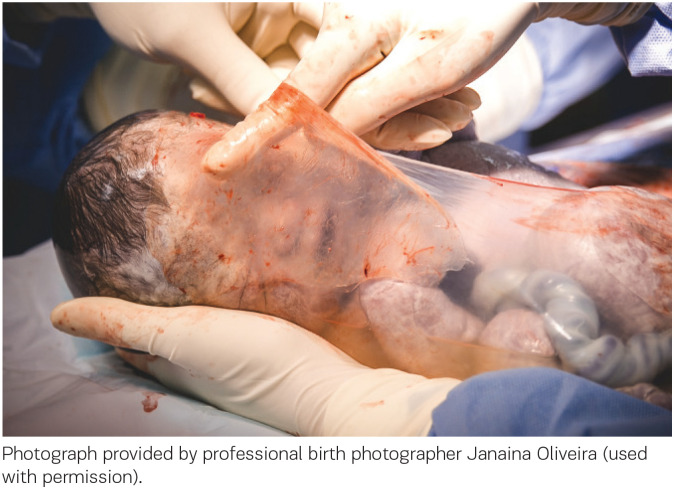
A newborn delivered en caul; the amniotic sac is opened after birth

Despite its cultural relevance, caul birth remains underexplored in research^5^. Reported prevalence estimates of caul birth vary widely in the literature, ranging from approximately 1 in 1000 to 1 in 80000 births. However, these estimates appear to be repeatedly cited across secondary sources, and the original empirical basis for these figures is not clearly documented. A previous qualitative study on amniotomy^6^, based on interviews with experienced midwives, found that midwives primarily expressed reluctance to perform amniotomy in order to avoid disrupting the physiological process of labor. The midwives viewed the intact membranes as a safety, since the whole ‘bag of waters’ surrounding the baby acts as a cushion. Caul birth was described by some of the midwives as the most natural birth, a utopia, and a constant aim when assisting women in labor^6^. Existing research on caul birth primarily focuses on neonatal outcomes in preterm cesarean sections (CS), with several studies suggesting that leaving the membranes intact and delivering the entire amniotic sac may reduce neonatal trauma due to the cushioning effect of the amniotic fluid^1,5,7^. A method for performing CS with the amniotic sac left intact was proposed by Abouzeid and Thornton^5^ in a study published in 1998. They described making a generous lower segment or classical uterine incision without rupturing the membranes, allowing the surgeon to insert a hand and bluntly separate the sac from the uterine wall. With the aid of fundal pressure, the intact sac – along with the placenta – is delivered as a whole and then opened on the mother’s thighs (Figure 3).

The authors also highlighted a potential risk of fetal hemorrhage if placental vessels are damaged during the procedure^5^. To reduce the risk of hemorrhage, researchers have proposed a modified technique in which only the initial portion of the intact amniotic sac is delivered through the uterine incision, the membranes are then immediately ruptured, and the baby is delivered. Their study found that maternal blood loss was comparable between caul CS and conventional CS, while the rate of asphyxia was significantly lower among preterm infants delivered using the caul method compared to control cases^7^. According to other researchers, the limited number of studies on caul births may contribute to a lack of training opportunities for obstetricians to perform CS while preserving the amniotic sac^1^.

The World Health Organization (WHO) states that most pregnancies are low-risk, yet medical interventions during labor and birth continue to rise – even in spontaneous, uncomplicated cases^8^. This trend has raised concerns about the overuse of intrapartum interventions, which may undermine women’s autonomy and diminish support for physiological birth^8,9^. The normalization of such practices has also influenced how physiological birth is defined and experienced^10^. In response, the WHO and recent studies emphasize the importance of supporting physiological birth to reduce unnecessary interventions and improve maternal and neonatal outcomes^8,10^. Evidence suggests that while current intrapartum care research often prioritizes risk avoidance, this approach can limit understanding and appreciation of the normal physiological processes of labor and birth^9,11^. During labor, the membranes may rupture spontaneously, or the care provider may choose to perform an amniotomy – or refrain from doing so. When amniotomy is withheld, caul birth remains a possible outcome. There is a notable lack of research on the prevalence, predictors, and outcomes of caul birth, despite its potential relevance to physiological birth. Addressing this gap is crucial for advancing research on physiological birth. For midwives, a thorough understanding of both the physiological and clinical dimensions of caul birth is essential to ensure safe care and support informed decision-making during labor and birth8,9,11.

The aim of this retrospective cohort study was to investigate the prevalence of caul birth, its predictors, and associated neonatal and maternal outcomes in a Swedish hospital setting.

## METHODS

This retrospective cohort study was based on a review of medical records from all births at one Swedish hospital during 2023. A one-year study period was selected to include all births occurring at the hospital during a complete calendar year and thereby capture all available cases of this rare outcome. Because the prevalence of caul birth has not been established in previous epidemiological studies, an *a priori* power calculation was not feasible. The study should therefore be regarded as exploratory.

### Setting

In 2023, a total of 98331 births occurred in Sweden^12^, and almost all women gave birth in hospitals, a service provided by the state-driven healthcare. In Sweden, midwives are the primary caregivers for intrapartum care when pregnancies and births are healthy, without medical complications. If complications occur during labor, midwives work in collaboration with obstetricians. The data collection for the present study was performed in one hospital in Sweden by reviewing electronic medical records of all births between 1 January and 31 December 2023. The included hospital, which manages low- and high-risk births, had 1382 births during 2023. Rates of spontaneous vaginal births at the unit were 76.3%, instrumental births 7.5%, and CS (elective and emergency) 16.2%. These rates are comparable with the overall rates in Sweden in 2023, which were 73.0% for spontaneous vaginal births, 7.5% for instrumental births, and 19.5% for CS^12^. The general clinical practice for amniotomy in Sweden, which is also followed at the selected delivery unit, is that it should be performed only when clinically indicated, i.e. in cases of labor dystocia, for induction of labor, or when internal fetal monitoring is required. Amniotomy is used in 40% of all births in Sweden^13^.

### Participants

**Figure 4:**
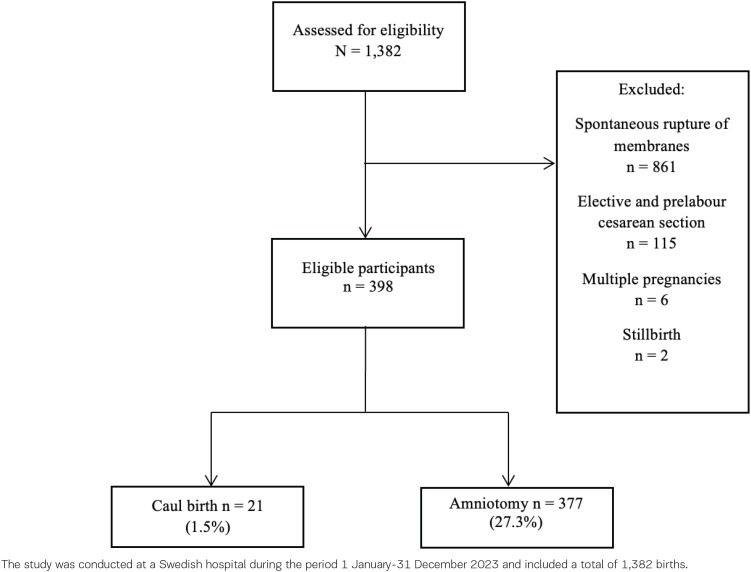
Flowchart of the study population.

The inclusion criteria were nulliparous and multiparous women with singleton pregnancies, with spontaneous or induced labor onset, who had a caul birth or an amniotomy performed during labor. Women with a spontaneous rupture of the membranes, elective and pre-labor CS, and those with stillbirth were excluded (Figure 4). Women with spontaneous rupture of membranes were excluded because the study aimed to compare caul birth with amniotomy, a clinical intervention that can be performed or withheld during labor.

### Data collection and variable definition

The data were retrieved from medical charts and registered on a specific report form developed by the researcher. The caul birth was identified as the absence of both amniotomy and spontaneous rupture of membranes, combined with explicit documentation by the midwife stating a caul birth occurred. Besides caul birth and amniotomy, data on the following parameters were collected: maternal body mass index (BMI) in the first trimester, parity, maternal age, and gestational age at birth. Furthermore, time for admission to the delivery unit, onset of labor (spontaneous, induction), use of epidural, time of birth and mode of birth, and maternal birth experience were selected. Finally, the neonatal outcomes, Apgar score and umbilical cord pH, were selected.

Maternal age was categorized as <30 and ≥30 years. Maternal BMI was categorized into the World Health Organization standards^14^; however, underweight and normal weight were combined, as no woman with a caul birth was underweight according to BMI. Gestational age was categorized as preterm (<37 weeks), term (37–42 weeks), and post-term (>42 weeks). The cutoff for umbilical cord arterial pH was set at <7.20 versus ≥7.20, as no infant in the caul birth group had a pH value below 7.10. Thresholds for defining abnormal umbilical cord pH vary between 7.20 and 7.00, and a pH value below 7.20 is often classified as mild acidemia, despite many neonates in this range being clinically vigorous^15^. A numerical rating scale was used to measure the birth experience. The woman rated her overall experience of the birth, with 0 signifying the worst imaginable experience and 10 the best imaginable experience. The rating was made before discharge from the hospital. The outcome variables were compared between women with caul birth and those with amniotomy. Data were complete for the vast majority of variables, and where data were missing, this is indicated in Table 1. Analyses were based on available data, and no imputation of missing values was performed. The STROBE guidelines for cohort studies were followed for this manuscript^16^.

**Table 1:** Maternal, obstetric and neonatal characteristics of the study population and by group, with statistical comparisons, a retrospective cohort study conducted at a Swedish hospital during 2023 (N=1382 births)

*Characteristics*	*Total population* *n (%)*	*Caul births* *n (%)*	*Amniotomy* *n (%)*	*p*
**Total**, n	398	21	377	
**Maternal age** (years), mean (SD)	30.3 (4.7)	30.5 (2.8)	30.2 (4.7)	0.397^a^
**BMI** (kg/m^2^), mean (SD)	26.3 (5.2)	26.3 (3.4)	26.3 (5.3)	0.500^a^
Missing, n	9	0	9	
**Parity**				<0.001^b^
0	192 (48.2)	1 (4.8)	191 (50.7)	
1	132 (33.2)	11 (52.4)	121 (32.1)	
2	52 (13.1)	7 (33.3)	45 (11.9)	
3	9 (2.2)	2 (9.5)	7 (1.9)	
≥4	13 (3.3)	0 (0)	13 (3.4)	
**Induction of labor**	185 (46.5)	1 (4.8)	184 (48.8)	<0.001^b^
**Epidural**	274 (68.8)	2 (9.5)	272 (72.1)	<0.001^b^
**Gestational age** (weeks)				0.192^c^
<37	6 (1.5)	1 (4.8)	5 (1.3)	
37–42	385 (96.7)	20 (95.2)	365 (96.8)	
>42	7 (1.8)	0 (0)	7 (1.9)	
**Admission to birth** (hours), median (IQR)	14.1 (7.1–27.0)	1.7 (0.8–3.5)	14.7 (8.3–28.1)	<0.001^c^
**Mode of birth**				0.119^b^
Spontaneously vaginal	329 (82.7)	21 (100)	308 (81.7)	
Instrumental	38 (9.5)	0 (0)	38 (10.1)	
Emergency cesarean section	31 (7.8)	0 (0)	31 (8.2)	
**Apgar score**				
1 minute, median (IQR)	9 (9–10)	9 (9–10)	9 (9–10)	0.637^c^
<7 at 5 minutes	9 (2.3)	0 (0)	9 (2.4)	1.000^b^
**Umbilical cord pH**				
Arterial, median (IQR)	7.21 (7.15–2.26)	7.28 (7.19–7.29)	7.21 (7.15–7.25)	0.091^c^
Missing, n	95	9	86	
Venous, median (IQR)	7.31 (7.27–7.35)	7.32 (7.28–7.38)	7.31 (7.27–7.25)	0.200^c^
Missing, n	17	6	11	
**Birth experience** (VAS), median (IQR)	8 (7–10)	9 (8.5–10)	8 (7–10)	0.024^c^
Missing, n	44	1	43	

VAS: visual analogue scale. IQR: interquartile range.

a t-test.

b Fisher’s exact.

c Mann-Whitney U test. Statistical significance was set at p<0.05.

### Ethics

The study followed the principles of the Declaration of Helsinki^17^. Ethical approval was obtained from the Swedish Ethical Review Authority (Approval number: 2019-03626; Date: 3 July 2019). (Approval number 2020-04657; Date: 25 September 2020) and (Approval number: 2024-00809-02; Date: 13 February 2024).

### Data analysis

Means and standard deviations or medians and interquartile ranges are used to present the continuous variables; categorical data are presented as absolute and relative frequencies. An independent-samples t-test or a Mann–Whitney U-test was used to compare the continuous background variables between women with caul birth and with amniotomy. For categorical variables, Fisher’s exact test was used instead of the Pearson chi-squared test due to small cell counts and unbalanced group sizes.

The potential predictors and outcomes were examined by simple binary logistic regression analyses, with caul birth as the outcome variable. Subsequently, to assess the independent effect of each predictor or outcome, multiple binary logistic regression analyses were conducted. Variables included in the multivariable logistic regression models were selected *a priori* based on clinical relevance and existing literature and not based on automated or purely statistical selection procedures. Data are presented as odds ratios (OR) with 95% confidence intervals (CI). The statistical significance was set overall at p<0.05. The analyses were carried out using STATA IC 16.0 (StataCorp LLC, College Station, TX, USA).

## RESULTS

### Sample characteristics

Caul birth was documented in 21 cases (1.5%) among the 1382 births at the hospital in 2023. The mean maternal age in the total cohort was 30.3 years (SD=4.7), and the mean BMI was 26.3 kg/m² (SD=5.2). Approximately half of the women underwent induction of labor (46.5%), and epidural analgesia was used in 68.8% of cases. The majority of births occurred at term (96.7%) and were spontaneous vaginal births (82.7%). The mean age and BMI for women with caul birth and those with amniotomy were similar. However, women with caul birth were significantly more often multiparous compared with women with amniotomy (p<0.001); only one nulliparous woman experienced a caul birth. The vast majority of women with caul birth (95.2%) had a spontaneous onset of labor, as only one had labor induced, compared with 48.8% of the women with amniotomy (p<0.001). Two women (9.5%) with caul birth used epidural analgesia, compared with 72.1% of the women who underwent amniotomy (p<0.001). The time spent in hospital before giving birth differed markedly between women with caul birth and those with amniotomy: 1.7 versus 14.7 hours (p<0.001). All women with caul birth had a spontaneous vaginal birth, and all but one gave birth at term. All neonates born in caul births had normal Apgar scores and umbilical cord pH levels. When compared with neonates born after amniotomy, both arterial and venous pH levels were slightly higher; however, these differences were not significant (p=0.091 and p=0.200, respectively). Women with caul birth reported a slightly higher median rating of their birth experience on the visual analog scale (9 vs 8), compared with women with amniotomy, a difference that was statistically significant (p=0.024). Maternal, obstetric, and neonatal characteristics are presented for the total study population and by group (caul birth and amniotomy) in Table 1.

### Factors associated with caul birth

The factors that remained statistically significant in the multiple logistic regression for caul birth were maternal overweight as assessed by BMI (OR=3.42; 95% CI: 1.10–10.64), the spontaneous onset of labor (OR=18.21; 95% CI: 2.31–143.50), and multiparity (OR=11.11; 95% CI: 1.34–92.10), all of which increased the odds of caul birth. The use of epidural analgesia decreased the odds of caul birth (OR=0.10; 95% CI: 0.02–0.44) (Table 2).

**Table 2:** Predictors associated with caul birth based on unadjusted and multiple binary logistic regression analyses, a retrospective cohort study conducted at a Swedish hospital during 2023 (N=1382 births)

*Variables*	*Unadjusted binary logistic regression*			*Multiple binary logistic regression*		
	*OR*	*95% CI*	*p*	*AOR*	*95% CI*	*p*
**Maternal age** (years)						
<30 (ref.)	1			1		
≥30	2.07	0.79–5.46	0.139	1.31	0.43–4.00	0.638
**BMI** (kg/m^2^)						
≤24.9 (ref.)	1			1		
25–29.9	2.87	1.03–7.74	0.044	3.42	1.10–10.64	0.034
≥30	0.87	0.21–3.55	0.844	1.21	0.26–5.71	0.807
**Gestational age** (weeks)	0.82	0.66–1.01	0.067	0.95	0.72–1.24	0.710
**Parity**						
Nulliparous (ref.)	1			1		
Multiparous	20.54	2.72–154.58	0.003	11.11	1.34–92.10	0.026
**Spontaneous onset of labor**						
No (ref.)	1			1		
Yes	19.1	2.53–143.52	0.004	18.21	2.31–143.50	0.006
**Epidural**						
No (ref.)	1			1		
Yes	0.40	0.01–0.18	<0.001	0.10	0.02–0.44	0.003

AOR: adjusted odds ratio. Statistical significance was set at p<0.05.

### Maternal and neonatal outcomes of caul birth

In the multiple logistic regression analysis, the only outcome that remained statistically significant for caul birth was the duration from hospital admission to birth, with longer duration associated with decreased odds of a caul birth (OR=0.63; 95% CI: 0.48–0.84). Regarding Apgar score at one minute, arterial pH <7.20, and birth experience according to VAS, no statistically significant associations were found (Table 3). Birth outcome (spontaneous vaginal, instrumental, emergency CS) was not included as an outcome variable in the multivariable regression analyses, as all women with caul birth had a spontaneous vaginal birth.

**Table 3:** Maternal and neonatal outcomes associated with caul birth based on unadjusted and multiple binary logistic regression analyses, a retrospective cohort study conducted at a Swedish hospital during 2023 (N=1382 births)

*Variables*	*Unadjusted binary logistic regression*			*Multiple binary logistic regression*		
	*OR*	*95% CI*	*p*	*AOR*	*95% CI*	*p*
**Admission to birth** (hours)	0.60	0.48–0.75	<0.001	0.63	0.48–0.84	0.002
**Apgar score 1 minute**	1.28	0.80–2.03	0.302	1.83	0.53–6.31	0.336
**Arterial umbilical cord pH**						
<7.20	0.65	0.16–2.67	0.555	2.74	0.48–15.59	0.257
≥7.20 (ref.)	1			1		
**Birth experience** (VAS)	1.30	0.94–1.80	0.106	1.08	0.64–1.83	0.761

VAS: visual analogue scale. AOR: adjusted odds ratio. Statistical significance was set at p<0.05.

## DISCUSSION

To our knowledge, this is the first Swedish study investigating the prevalence of caul birth, its predictors, and associated neonatal and maternal outcomes. The observed prevalence of 1.5% in this study indicates that caul birth, while still rare, may occur more frequently than previously reported in modern obstetric and midwifery practice. Various previous sources report the prevalence of caul birth as ranging between 0.1% and 0.00125%; however, despite extensive searches, the primary sources for these numbers could not be identified. This discrepancy highlights the need for more systematic data collection and clearer definitions when reporting caul births, in order to better understand their true frequency and clinical relevance.

In addition to estimating prevalence, this study also identified several factors that were significantly associated with the occurrence of caul birth. In the multiple binary regression analysis, spontaneous onset of labor and absence of epidural analgesia were found to be significant factors associated with caul birth. This finding aligns with previous research and current recommendations, which state that spontaneous onset of labor minimizes interventions and reduces the risk for over-medicalization of the physiological birth process^18-22^. Nevertheless, it is important to note that one woman with a caul birth had an induced labor, and two used epidural analgesia, which highlights the importance of individualized care during induction; further interventions such as amniotomy may not always be necessary once labor has been initiated.

Maternal overweight according to BMI was also identified as a significant factor associated with caul birth. Previous research has shown that women with higher BMI may have shorter labor durations, particularly during the second stage^23,24^. One possible explanation for the observed association is that a more rapid labor progression in these women reduces the likelihood of amniotomy being performed and thereby increases the chances of the amniotic sac remaining intact until birth. Multiparity was also identified as a significant predictor of caul birth. This is consistent with the understanding that multiparous women generally experience shorter labors and are more likely to have an uncomplicated course of labor, compared with nulliparous women^25-27^. However, it is noteworthy that one nulliparous woman in this study also experienced a caul birth, indicating that although less frequent, such occurrences are still possible among nulliparous women.

In addition to identifying associated factors, this study also explored outcomes associated with caul birth and found that all women with caul birth had a spontaneous vaginal birth. The neonatal outcomes associated with caul birth were uniformly reassuring. All 21 infants had normal Apgar scores and umbilical cord pH values, indicating no signs of immediate compromise. While the pH levels in both arterial and venous blood were slightly higher compared with those in the amniotomy group, the differences were not statistically significant. Umbilical cord pH data were missing for 9 of 21 arterial samples and 6 of 21 venous samples, which limits the analysis and interpretation of this outcome; this missingness is likely due to midwives refraining from collecting pH values in cases of uncomplicated births when the newborn was healthy and vigorous. In addition, the routine of collecting umbilical cord pH samples is debated among Swedish midwives and obstetricians, as some argue that such sampling may not be clinically useful for healthy, vigorous newborns and can disrupt early bonding. Despite these limitations, the observed trend may support the hypothesis that the intact amniotic sac provides a protective, cushioning effect during birth – a mechanism proposed in previous studies^1,6^. Importantly, no adverse neonatal outcomes were observed in the caul group, and the findings are consistent with current evidence and labor management guidelines regarding expectant management in uncomplicated labor^28-32^.

The only significant outcome in the multiple binary regression analysis was the duration from hospital admission to birth, with a shorter duration associated with increased odds of caul birth. This finding suggests that these labors may have progressed rapidly and/or that the women were admitted in advanced stages of labor. It can be hypothesized that women who remain at home during the early stages of labor are less likely to undergo interventions and more likely to experience a physiological birth. This observation serves as a reminder that while labor interventions can be beneficial and life-saving when indicated, their overuse may be unnecessary and potentially detrimental to the natural birth process^8,9^.

In the multiple binary regression analysis, positive maternal birth experience was not found to be a significant outcome of caul birth. However, women who gave birth with the membranes intact rated their birth experience significantly higher than those who underwent amniotomy, despite the numerical difference being relatively small (VAS: 9 vs 8). This statistically significant finding may reflect greater satisfaction commonly associated with low-intervention, spontaneous births^33,34^. According to Olza et al.^35^, women describe feelings of joy after achieving and experiencing physiological childbirth. Whether directly witnessed or conveyed by care providers, the knowledge of having experienced a caul birth might contribute to a more positive and empowering birth narrative. These results suggest that the symbolic significance of caul birth may still hold meaning for women giving birth today. In line with this, the International Code of Ethics for Midwives states that midwives should respond to the psychological, physical, emotional, and spiritual needs of women^36^. To further understand the experiences and perspectives of women who have had a caul birth, qualitative interview studies exploring women’s perceptions of caul birth and its influence on birth experience are needed.

### Strengths and limitations

A major strength of this study is the low level of missing data and the inclusion of a complete annual cohort of all births at the hospital. The manual chart review allowed for the collection of detailed clinical information and enhanced data validity compared to registry-based studies, reducing the risk of missing or misclassified cases due to absent diagnostic codes or coding errors. Furthermore, adjustments for several key confounders were made in the statistical analyses, strengthening the reliability of the observed associations.

This study has several limitations that should be acknowledged. First, its retrospective design introduces potential sources of bias, including reliance on clinical documentation. Caul births may have been underreported, as midwives may not have consistently recorded the presence of intact membranes at birth. This may have resulted in misclassification bias, whereby some caul births may have been classified as non-caul births. Such misclassification could have led to an underestimation of the prevalence of caul birth and may have attenuated the observed associations. Second, the study was conducted at a single hospital, which limits the generalizability of the findings. Including data from multiple centers would have provided a broader perspective and allowed for greater variation in clinical practice. Third, the overall number of caul births was relatively small (n=21), which limits the statistical power to detect differences – particularly for less frequent outcomes such as neonatal complications – and increases the risk of Type II errors. Furthermore, the limited sample size resulted in wide confidence intervals, which may indicate lower precision in the estimated effect sizes. Although adjustments were made for several relevant confounders, residual confounding cannot be excluded. Information on factors such as cervical dilatation at admission and fetal position was not available and may have influenced both the likelihood of amniotomy and the occurrence of caul birth. Confounding by indication should also be considered, as women who underwent amniotomy may have differed systematically from those who experienced a caul birth. Clinical factors contributing to the decision to perform amniotomy may also have influenced maternal and neonatal outcomes.

Furthermore, women with spontaneous rupture of membranes were excluded from the analyses because the study focused on comparing caul birth with amniotomy. As spontaneous rupture of membranes is common during labor, this exclusion may have introduced selection bias and resulted in a study population that is not fully representative of all laboring women.

Missing data were generally limited; however, umbilical cord pH values were missing for a substantial proportion of neonates, which may have reduced statistical power and limited the interpretation of neonatal outcomes. Consequently, the findings should be interpreted with caution. Finally, the study lacked information on the quality of the amniotic fluid. It remains unclear whether the presence of meconium-stained fluid within an intact sac affects neonatal wellbeing. Future multicenter studies should investigate neonatal outcomes among caul births with meconium-stained amniotic fluid to determine whether intact membranes influence neonatal adaptation or the need for intervention.

## CONCLUSIONS

This study is the first in Sweden to examine caul birth, confirming it as a rare but not insignificant event. Caul births were associated with spontaneous, low-intervention labor and showed no adverse neonatal outcomes. These findings support current recommendations against routine amniotomy in uncomplicated labor. Further research in larger study populations is needed to confirm these results.

## Supplementary Material



## Data Availability

The data supporting this research are available from the author on reasonable request.
